# Longitudinal genomic surveillance of multidrug-resistant *Escherichia coli* carriage in a long-term care facility in the United Kingdom

**DOI:** 10.1186/s13073-017-0457-6

**Published:** 2017-07-25

**Authors:** Hayley J. Brodrick, Kathy E. Raven, Teemu Kallonen, Dorota Jamrozy, Beth Blane, Nicholas M. Brown, Veronique Martin, M. Estée Török, Julian Parkhill, Sharon J. Peacock

**Affiliations:** 1 Department of Medicine, University of Cambridge, Box 157, Addenbrooke’s Hospital, Hills Road, Cambridge, CB2 0QQ UK; 2 Wellcome Trust Sanger Institute, Wellcome Genome Campus, Hinxton, Cambridge, CB10 1SA UK; 3 Cambridge Public Health England Microbiology and Public Health Laboratory, Box 236, Addenbrooke’s Hospital, Hills Road, Cambridge, CB2 0QQ UK; 4 British Society for Antimicrobial Chemotherapy, 53 Regent Place, Birmingham, B1 3NJ UK; 5 Department of Medical Microbiology, Pathology Sciences Building 1, Southmead Hospital, Bristol, BS10 5NB UK; 6 Cambridge University Hospitals NHS Foundation Trust, Hills Road, Cambridge, CB2 0QQ UK; 7 London School of Hygiene and Tropical Medicine, London, WC1E 7HT UK

**Keywords:** ESBL, ST131, Genome, Sequence, Phylogeny

## Abstract

**Background:**

Residents of long-term care facilities (LTCF) may have high carriage rates of multidrug-resistant pathogens, but are not currently included in surveillance programmes for antimicrobial resistance or healthcare-associated infections. Here, we describe the value derived from a longitudinal epidemiological and genomic surveillance study of drug-resistant *Escherichia coli* in a LTCF in the United Kingdom (UK).

**Methods:**

Forty-five of 90 (50%) residents were recruited and followed for six months in 2014. Participants were screened weekly for carriage of extended-spectrum beta-lactamase (ESBL) producing *E. coli*. Participants positive for ESBL *E. coli* were also screened for ESBL-negative *E. coli*. Phenotypic antibiotic susceptibility of *E. coli* was determined using the Vitek2 instrument and isolates were sequenced on an Illumina HiSeq2000 instrument. Information was collected on episodes of clinical infection and antibiotic consumption.

**Results:**

Seventeen of 45 participants (38%) carried ESBL *E. coli*. Twenty-three of the 45 participants (51%) had 63 documented episodes of clinical infection treated with antibiotics. Treatment with antibiotics was associated with higher risk of carrying ESBL *E. coli*. ESBL *E. coli* was mainly sequence type (ST)131 (16/17, 94%). Non-ESBL *E. coli* from these 17 cases was more genetically diverse, but ST131 was found in eight (47%) cases. Whole-genome analysis of 297 ST131 *E. coli* from the 17 cases demonstrated highly related strains from six participants, indicating acquisition from a common source or person-to-person transmission. Five participants carried highly related strains of both ESBL-positive and ESBL-negative ST131. Genome-based comparison of ST131 isolates from the LTCF study participants with ST131 associated with bloodstream infection at a nearby acute hospital and in hospitals across England revealed sharing of highly related lineages between the LTCF and a local hospital.

**Conclusions:**

This study demonstrates the power of genomic surveillance to detect multidrug-resistant pathogens and confirm their connectivity within a healthcare network.

**Electronic supplementary material:**

The online version of this article (doi:10.1186/s13073-017-0457-6) contains supplementary material, which is available to authorized users.

## Background

*Escherichia coli* is a leading cause of bloodstream and urinary tract infections [1, 2]. In the United Kingdom (UK), there has been an increase in *E. coli* bloodstream infections since 2008, with a 16.7% rise in cases between 2010 and 2014 [3]. The uropathogenic *E. coli* (UPEC) lineage sequence type (ST)131 was initially reported in several countries during 2008 [4–6] and has since become widely disseminated. *E. coli* ST131 frequently carries plasmid-mediated extended-spectrum beta-lactamase (ESBL) genes that confer resistance to third-generation cephalosporins [7–9]. Infection with ESBL *E. coli* is associated with increased hospital stay, healthcare costs and mortality compared with infections caused by non-ESBL *E. coli* [10–12]. The most frequently identified ESBL gene in *E. coli* ST131, *bla*_CTX-M-15_, is globally disseminated and predominates in this ST in North America [6, 7], the UK [13], Europe [14–16], Asia [17, 18] and South America [19]. *E. coli* bloodstream isolates submitted between 2001 and 2010 to the British Society for Antimicrobial Chemotherapy (BSAC) Bacteraemia Resistance Surveillance Programme demonstrate the prevalence of this lineage within the UK [20]. Clonal complex (CC) 131 accounted for 12% of isolates and contained 81.7% of all *bla*CTX-M-1 group (which includes *bla*_CTX-M-15_) genes [20].

Global surveillance of antibiotic consumption and the emergence of resistance is gathering pace through numerous initiatives, including those by the World Health Organization (WHO), European Centre for Disease Prevention and Control (ECDC) and the Department of Health [21–25]. Target species include *E. coli*, particularly *E. coli* that are resistant to third-generation cephalosporins and fluoroquinolones. The development of surveillance frameworks requires consideration of the target populations.

The global increase in life expectancy has been associated with a rise in the number of people requiring care in long-term care facilities (LTCFs). LTCFs also provide increasing levels of post-acute, rehabilitative and palliative care to optimise patient flow through acute hospitals [26, 27]. Point prevalence studies have reported carriage rates of multidrug-resistant *E. coli* in residents of LTCF in excess of 50% in Ireland and 40% in the UK [28, 29]. This is significantly higher than the general population, with a recent meta-analysis of community carriage rates reporting a pooled prevalence of ESBL carriage of 2% in the Americas, 4% in Europe, 15% in the eastern Mediterranean and 22% in South East Asia and Africa [30].

Although high rates of ESBL *E. coli* in LTCFs have been established, genomic characterisation of the associated isolates has been limited and published studies have utilised molecular techniques such as pulsed-field gel electrophoresis and polymerase chain reaction (PCR)-based assays [28, 29, 31–33], which lack the discrimination of whole-genome sequencing (WGS). Here, we describe a longitudinal study of ESBL-*E. coli* carriage by residents of a LTCF, in which we determine the frequency of *E. coli* ST131 isolates and of non-ESBL *E. coli* isolates in the same patients. Analysis of WGS data for 399 *E. coli* isolates provided a detailed genetic understanding of the relationships between ESBL-positive and ESBL-negative *E. coli* within and between study participants. We extended this analysis by comparing 297 ST131 study genomes with more than 200 ST131 genomes of bacteria associated with bloodstream infection in patients across England to place our LTCF isolates into a broader genetic context.

## Methods

### Study design, setting and participants

A prospective observational cohort study was conducted during a six-month period in 2014 at a LTCF in Cambridgeshire in the UK, details of which have been published previously in relation to the study of *Enterococcus faecium* [34]. In brief, the LTCF had 105 beds and was sub-divided into five separate units to which residents were assigned based on cognitive impairment and physical disability.

### Sampling, microbiology and data collection

All residents admitted to the LTCF during the study period were eligible for inclusion. Residents were excluded if they refused consent, were on an end-of-life care pathway or were strongly resistant to basic personal care. Healthcare staff collected stool and urine specimens weekly from study participants, which were processed within 24 h (48 h at weekends). A total of 10 μl of each sample was plated either directly (urine samples only) or following overnight enrichment in 5 mL of Tryptic Soy broth (Sigma-Aldrich, St Louis, MO, USA) supplemented with cefpodoxime (Oxoid, Basingstoke, UK) at 1 μg/mL onto Brilliance ESBL agar (Oxoid, Basingstoke, UK) and incubated at 37 °C in air for 24 h. Putative ESBL *E. coli* colonies based on colony colour on chromogenic agar were speciated using matrix-assisted laser desorption/ionization time-of-flight mass spectrometry (MALDI-TOF) (Bruker Daltoniks, Bremen, Germany). A single colony from each positive sample was taken forward for further testing. Antimicrobial susceptibility was determined using the Vitek2 instrument (BioMérieux, Marcy l’Etoile, France) with the N206 card. Expression of ESBL was confirmed using the ESBL and AmpC Detection Disc Set (D68C1, Mast Group, Bootle, UK). All stools positive for ESBL-*E. coli* were cultured for non-ESBL *E. coli* by plating 10 μL of stool onto Brilliance UTI agar (Oxoid, Basingstoke, UK) and incubating at 37 °C in air for 24 h. Presumptive *E. coli* colonies were sub-cultured onto Columbia Blood Agar with the addition of a 10 μg cefpodoxime disc (Oxoid, Basingstoke, UK). Colonies growing at the edge of the zone of inhibition were selected for identification and antimicrobial susceptibility testing as above. Data were collected from participant nursing care plans and medical records on episodes of infection and antimicrobial consumption. Statistical analysis was performed using STATA v13.1 (STATA, College Station, TX, USA).

### Bacterial sequencing and analysis

Genomic DNA was extracted from single colonies using the QIAxtractor (QIAgen, Hilden, Germany). Library preparation was conducted according to the Illumina protocol, and sequencing was performed on an Illumina HiSeq2000 with 100-cycle paired-end runs. Ninety-six samples were multiplexed per lane to give an average depth of coverage of ~90-fold. Sequence data have been submitted to the European Nucleotide Archive (ENA) under the accession numbers listed in Additional file 1.

Sequence reads were assembled using Velvet v1.2 [35] and VelvetOptimser v2.2.5 (http://www.vicbioinformatics.com/software.velvetoptimiser.shtml). Assembly improvement was performed using the assembly with the best N50 and SSPACE was used for contig scaffolding [36]. GapFiller was used to close sequence gaps [37] and annotation was performed using PROKKA v1.11 [38] and a genus specific database from RefSeq [39]. STs were identified from the sequence data using the Warwick MLST database [40] and an in-house script [41, 42]. Sequence reads for ST131 isolates were mapped to the *E. coli* reference genome NCTC13441 (European Nucleotide Archive [ENA] accession number ERS530440) using SMALT v0.7.4 [43]. Variants were detected using samtools mpileup v0.1.19 [44] and the parameters ‘-d 1000 –DsugBf’ and bcftools v0.1.19, giving a BCF file of all variant sites. A variant quality score of greater than 50 and mapping quality of greater than 30 was used. The majority base call was required to be present in more than 75% of reads with a minimum mapping of four reads, with at least two mapping to each strand. A pseudo-genome was created by substituting bases called at each site in the BCF file into the reference genome. Any sites deemed uncertain following quality scoring were substituted with an N, along with any deletions identified in the context of the reference genome. To create a ‘core’ genome, mobile genetic regions were masked if they were annotated as predicted phage-, plasmid-, insertion sequence (IS)- or transposon-related genes, or if PHAST identified a putative prophage [45]. Gubbins was used to identify and remove recombination within each genome, giving the final ‘core’ genome [46], and maximum likelihood phylogenies were created using RAxML with 100 bootstraps and a mid-point root [47]. Trees were visualised using FigTree (v1.4.2) [48] and iTOL (v3) [48, 49].

The presence of antimicrobial resistance genes was determined by comparison of genomes to an in-house database using ResFinder [50]. *fimH* alleles were identified using in silico PCR and primers detailed in Weissman et al. [51] and Colpan et al. [52]. Seaview was used to curate and assign *fimH* types, detect fluroquinolone resistance mutations and SNPs associated with C0 and C2 [53]. Analysis of the accessory (non-core) genome composition was conducted as described previously [54]. Distribution of a mobile genetic element (MGE) among the isolates was determined through assembly alignment with MUMmer [55] and with sequence read mapping using SRST 2 [56].

Two further whole-genome sequence datasets were retrieved from the European Nucleotide Archive: (1) 75 ST131 isolates associated with bloodstream infection in patients at the Cambridge University Hospitals NHS Foundation Trust between 2006-2012 (Project PRJEB4681); and (2) ST131 isolates associated with bloodstream infection in ten hospitals in England between 2001 and 2011, submitted to the British Society for Antimicrobial Chemotherapy Resistance Surveillance Project (n = 146, Project PRJEB4681).

## Results

### Study participants

Forty-five of 90 (50%) eligible residents were recruited to the study. The median age of study participants was 82 years (range = 40–104 years, interquartile range [IQR] = 71–87 years), and 29 (64%) were women. Three participants were lost to follow-up because of death (n = 2) or transfer elsewhere (n = 1). The median duration of residence in the LTCF by the time the study began was 16 months (range = 5 days–54 months, IQR = 6–41 months). Twenty-nine percent (n = 13) of recruited residents had the capacity to consent for themselves to take part. The remaining 71% (n = 32) were recruited following discussion with a resident’s consultee who considered whether the resident in question would agree themselves to take part if they had the capacity to do so. Stool samples were collected at recruitment and then at least one week apart until the end of the study period, discharge from the LTCF or death.

### Infective episodes and antibiotic consumption

Debilitated patients are more prone to bacterial infection and antibiotic consumption selects for antibiotic resistant bacteria. In light of this, we collected information on episodes of infection and antibiotic consumption during the study and antibiotic consumption in the 12-month period preceding this. During the study, 23/45 (51%) participants had 63 documented infective episodes (median = 1, range = 1–5, IQR = 1–3.5). Infections of the urinary tract were the most common (n = 33, 54%), followed by those affecting the respiratory tract (n = 16) and skin and soft tissue (n = 8). The focus of infection was not specified in four cases. Diagnoses were based on clinical features alone with the exception of urinary tract infections, which were investigated using urinalysis to detect leucocytes and nitrites. No clinical specimens were collected from the study cohort for microbiological culture. All episodes were treated with at least one course of empiric antibiotics (median = 2 courses per patient, range = 1–6, IQR = 1–4). The most frequent antibiotics used were trimethoprim (16/63 infective episodes, 25%), co-amoxiclav (14/63, 22%) and flucloxacillin (10/63, 16%). In addition, two catheterised residents (participants P4 and P6) were on long-term prophylactic antibiotics (trimethoprim and metronidazole, respectively). Antibiotic consumption was also common in the 12 months prior to enrolment, with 31/45 (68.9%) participants receiving a total of 83 courses of antibiotics (median = 3, range = 1–5, IQR = 1–4). The three most frequently prescribed antibiotics prior to enrolment were trimethoprim (23/83, 28%), co-amoxiclav (21/83, 25%) and amoxicillin (11/83, 13%).

### *E. coli* carriage

**Fig. 1:**
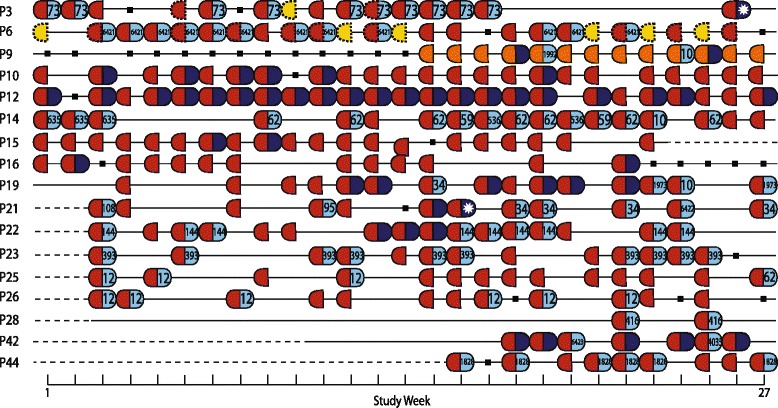
Timeline of results for ESBL-positive *E. coli* participants. Each row represents an ESBL-positive *E. coli* participant (P) and their culture results for ESBL *E. coli* and non-ESBL *E. coli* over 27 weeks. Each positive week is shown by a capsule of two halves, the *left* side representing isolation of ESBL *E. coli* and the *right* side non-ESBL *E. coli*. Results shown are for stool culture, with the exception of *yellow dotted half-capsules*, which represent ESBL-positive *E. coli* catheter urine (P3 and P6), and *red dotted half-capsules* (P3 and P6), which represent a positive stool and urine sample in the same week. Capsules are *coloured* by ESBL *E. coli* (*red*: ST131 ESBL *E. coli*, *orange*: ST38 ESBL-*E. coli*) and non-ESBL *E. coli* (*dark blue*: ST131 non-ESBL *E. coli*; *light blue*: non-ST131 non-ESBL *E. coli*). *White asterisk*, ST not known as sequencing failed to generate high quality data; *black square*, sample taken but no *E. coli* isolated; *dashed line*, not yet recruited into the study or no longer enrolled. Numbers shown for non-ESBL *E. coli* represent MLST sequence type

ESBL *E. coli* was cultured from stool from 17/45 participants (38%) (isolated from 241/691 stool samples tested), none of whom were known previously to be ESBL *E. coli* carriers. Figure 1 shows the timeline for positive and negative samples. Most ESBL-positive *E. coli* participants were positive on the first stool tested and were carriers thereafter. The exceptions were P9, who became positive after 13 negative samples, and P16, who became negative after 13 ESBL-positive *E. coli* samples. Urine was also cultured if a urinary catheter was present. Two of the three catheterised participants (P3 and P6) had ESBL *E. coli* isolated from urine (3/4 samples and 18/18 samples, respectively), both of whom carried ESBL *E. coli* in stool. The third case (P4) had ESBL-negative *E. coli* in both urine and stool.

Almost one-half (7/17, 41%) of ESBL-positive *E. coli* participants lived in unit 3, which provided residential and minor nursing care for residents with dementia. The majority (15/17, 88%) of ESBL-positive *E. coli* participants had received at least one course of antibiotics in the 12 months prior to study enrolment, compared with 16/28 (57%) participants who were ESBL-negative. Antibiotic consumption on at least one occasion was associated with higher risk of having ESBL *E. coli* isolated from stool (odds ratio [OR] = 5.6, 95% confidence interval [CI] = 1.1–29.4, *p* = 0.04 – logistic regression model).

All 241 stools positive for ESBL *E. coli* were also cultured for non-ESBL *E. coli*). At least one isolate was cultured from all 17 ESBL-positive *E. coli* participants (isolated from 139/243 stools).

### Characterising *E. coli* isolates by MLST and ESBL encoding genes

We sequenced 401 *E. coli* isolates (241 ESBL-*E. coli* from stool, 21 ESBL-*E. coli* from urine and 139 non-ESBL *E. coli* from stool). Two non-ESBL *E. coli* genomes were excluded from further analysis based on inadequate quality of sequence data. STs were identified from sequence data for the 399 remaining isolates. The 262 ESBL *E. coli* isolates were assigned to ST131 (n = 249) or ST38 (n = 13). Sixteen participants carried ST131 ESBL *E. coli* and the remaining participant (P9) carried ST38 ESBL *E. coli* (Fig. 1). Non-ESBL *E. coli* were more genetically diverse, with 21 STs identified among the 137 isolates. The most common ST for non-ESBL *E. coli* was also ST131 (n = 48, 35%), which was carried by 9/17 participants, eight of whom were also positive for ST131 ESBL *E. coli* (Fig. 1). Seven participants were positive for more than one ST (median = 1 ST, range = 1–5 STs) and five STs were carried by more than one participant (ST10, ST12, ST34, ST62 and ST131) (Fig. 1). ESBL was encoded by *bla*_CTX-M-15_ in all 262 ESBL *E. coli* isolates.

### Genomic focus on *E. coli* ST131 from LTCF participants

**Fig. 2:**
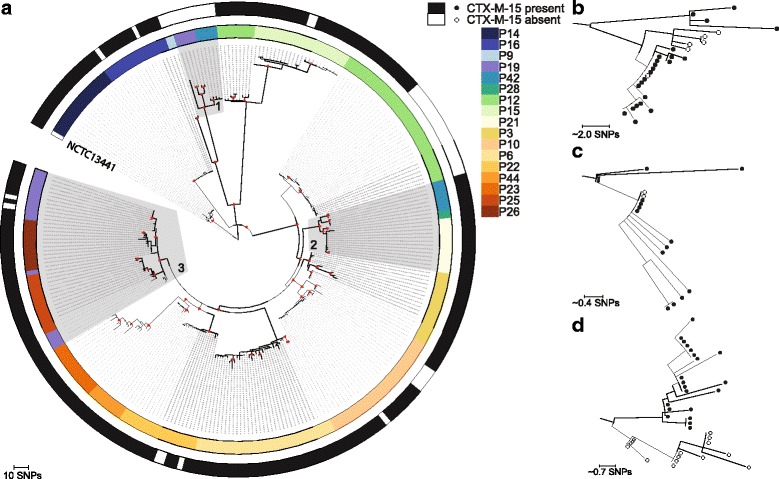
Phylogeny of ST131 isolates from study participants. **a** Mid-point rooted maximum likelihood tree based on the core genome of 297 ST131 isolates from 17 participants and the reference *E. coli* NCTC13441 genome after removal of MGEs and recombination events. The *inner ring* provides a link between each isolate genome and the participant from which this was cultured. The *outer ring* shows the presence of *bla*_CTX-M-15_, expression of which results in an ESBL phenotype. *Grey shaded blocks* highlight three clades of closely related isolates from multiple participants. *Red triangles* indicate nodes with bootstrap values of more than 90%. **b**, **c**, **d** Isolates from P10, P22 and P12, respectively

A maximum likelihood tree based on 797 single nucleotide polymorphisms (SNPs) in the core genome of 297 ST131 (see Additional file 1 for sequencing quality data) isolates from 17 participants compared with the reference *E. coli* NCTC13441 genome is shown in Fig. 2a. Isolates were distributed into multiple highly related clades, each corresponding to a positive participant. The pairwise SNP difference for isolates within each clade was in the range of 0–12 SNPs (median = 4 SNPs). Based on the upper limit of 12 SNPs for within-host diversity, three groups of participants were defined as carrying the same clade: P19 and P42; P21, P28 and P42; and P19, P25 and P26, labelled as 1, 2 and 3, respectively in Fig. 2a. These six participants all resided in unit 3. Additionally, three participants (P19, P12 and P42) each carried two distinct ST131 clades. In each case, these were deemed to be independent based on their genetic distance (more than 100 SNPs apart in each case) and position in the phylogenetic tree.

**Fig. 3:**
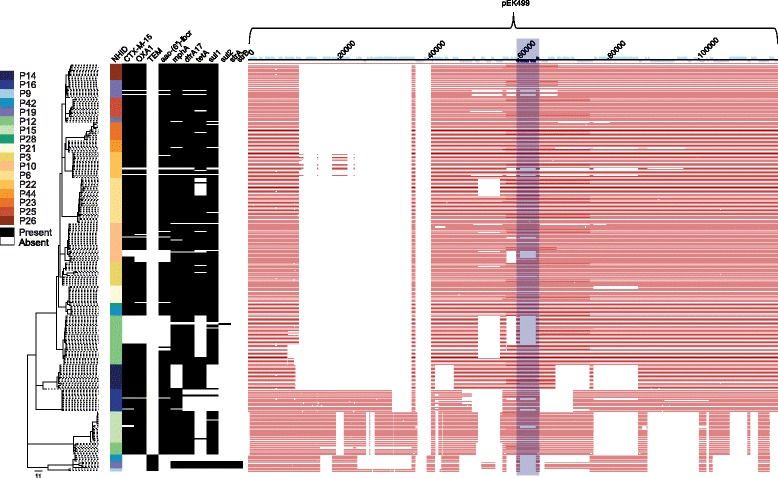
Antimicrobial resistance genes present in LTCF ST131 isolates and results of mapping to plasmid pEK499. Mid-point rooted maximum likelihood tree based on the core genome of 297 ST131 isolates from 17 participants and the reference *E. coli* NCTC13441 genome after removal of MGEs and recombination events. The first *vertical coloured column* links genomes to study participant. Antimicrobial resistance genes are shown as *black* (present) or *white* (absent). *blaOXA1* beta-lactam, *aac-(6’)-Ib-cr* aminoglycoside, *mphA* macrolide *dfrA17* trimethoprim, *tetA* tetracycline, *sul1*/*sul2* sulphonamide, *strA*/*strB* streptomycin resistance genes. Fragments of the pEK499 plasmid that are shared with a corresponding isolate are shown as *red horizontal blocks*. The *bla*_CTX-M-15_ cassette (the *bla*_CTX-M-15_ gene together with the flanking IS elements) is highlighted in *dark blue*

Of the 16 participants who carried ESBL *E. coli* ST131, 11 carried only ST131 ESBL *E. coli* and eight carried a mixture of ST131 ESBL *E. coli* and ST131 non-ESBL *E. coli*. In addition, P9 carried non-ESBL *E. coli* ST131 in combination with ST38 ESBL *E. coli*. The degree of within-host relatedness between ESBL *E. coli* and non-ESBL *E. coli* ST131 was illustrated by annotating the tree for the presence of *bla*_CTX-M-15_ (Fig. 2a), which revealed two patterns. Participants P10, P15, P16, P19 and P22 each carried clades that contained a mixture of highly related ESBL *E. coli* and non-ESBL *E. coli* (see Fig. 2b and c for examples based on genomes from P10 and P22). By contrast, participant P19 carried genetically distinct ESBL *E. coli* and non-ESBL *E. coli* ST131 clades, which may be indicative of failure of *bla*_CTX-M-15_ to transfer in vivo from one clade to the other. P12 appeared to display carriage of both patterns and carried two distinct clades, one consisting of only isolates harbouring *bla*_CTX-M-15_ and a second clade containing both highly related ESBL and non-ESBL *E. coli* (Fig. 2d). Variation in the presence of beta-lactam (*bla*_OXA-1_ *)*, aminoglycoside (*aac-(6’)-Ib-cr)*, macrolide (*mphA)*, trimethoprim (*dfrA17)*, tetracycline (*tetA)*, sulphonamide (*sul1* and *sul2)* and streptomycin (*strA* and *strB)* resistance genes (Fig. 3) was also observed (Additional file 1). In five clades, loss/gain of other genes was associated with *bla*_CTX-M-15_, which is consistent with these residing on the same MGE. Analysis of the accessory (non-core) genome composition was performed to examine the context of the *bla*_CTX-M-15_ gene in ESBL *E. coli* isolates. In all *bla*_CTX-M-15_ positive isolates, the gene was associated with an MGE that resembled the peK499 plasmid [9], a hybrid of Incompatibility Type F replicons FIA and FII. The ESBL *E. coli* isolates revealed several variants of the peK499-like plasmid, as shown by the variable coverage after aligning the whole genome sequences against the peK499 plasmid (Fig. 3). Sequence coverage of the plasmid among the *bla*_CTX-M-15_ positive isolates was in the range of 54–83% (median = 76%). Interestingly, in non-ESBL *E. coli* isolates, carriage of peK499-like plasmid sequences that lacked a region containing the *bla*_CTX-M-15_ gene was also observed. In the peK499 plasmid, *bla*_CTX-M-15_ is flanked on both sides by insertion sequence (IS26) transposase genes, suggesting a highly mobile cassette that may explain the loss and gain of ESBL status in the highly related ST131 isolates.

### Relatedness between ST131 from the LTCF and other healthcare settings

**Fig. 4:**
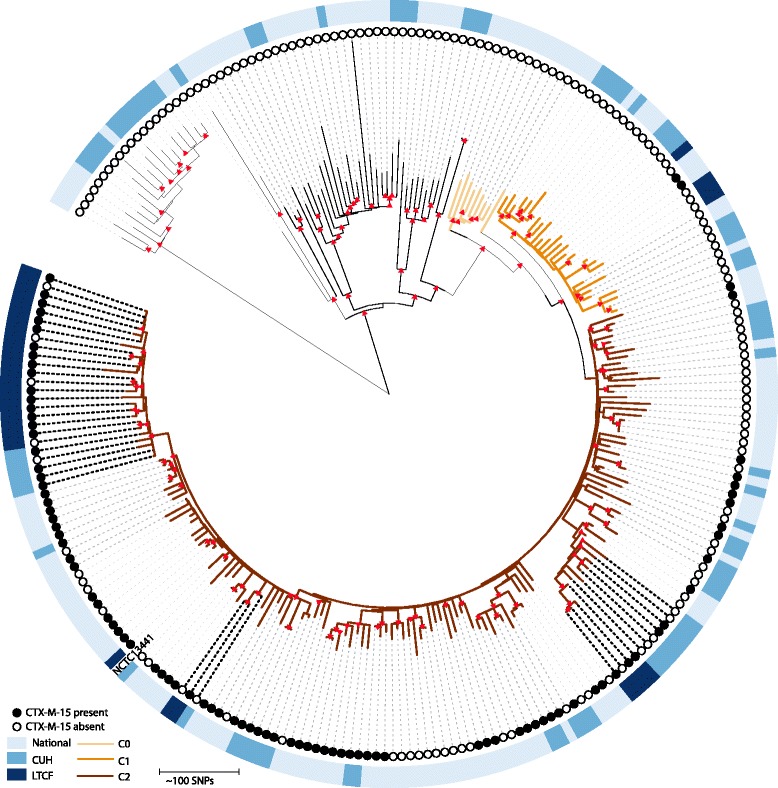
Phylogeny of representative LTCF ST131 isolates and ST131 isolates from CUH and a national collection. Mid-point rooted maximum likelihood tree of the core genome of 30 ST131 isolates from the LTCF and 221 ST131 isolates associated with bloodstream infection at the nearest acute hospital (CUH) and nationally following removal of MGEs and recombination events. The *outer ring* shows the origin of each isolate. *Black dashed lines* highlight three highly related clusters of LTCF and CUH isolates. *Filled red triangles* indicate nodes with bootstrap values of more than 90%. *Coloured branches* demonstrate the sub-clade divisions

Thirteen of the 17 ESBL-positive *E. coli* participants were admitted to the LTCF from the Cambridge University Hospitals NHS Foundation Trust (CUH) in the year prior to enrolment or during the study period. To determine the genetic relatedness between the study ST131 isolates and ST131 from patients at this hospital and further afield in England, we combined study genomes with whole-genome sequence data for 75 ST131 isolates (22 ESBL *E. coli*, 53 non-ESBL *E. coli*) from CUH and 146 ST131 isolates (52 ESBL *E. coli*, 94 non-ESBL *E. coli*) from ten hospitals across England, all associated with bloodstream infection. A subset of 30 study participant isolates were included in the analysis, consisting of one each of ESBL *E. coli* and non-ESBL *E. coli* from each lineage carried by participants. Data for these 251 ST131 isolates were used to construct a maximum likelihood tree based on 7676 SNPs in the core genome (Fig. 4). In this broader genetic context, isolates from LTCF participants fell into six clusters that were interspersed throughout the tree, although the majority (20/30, 16 ESBL *E. coli* and four non-ESBL *E. coli*) resided in a single cluster containing isolates from 12 participants. The remaining clusters contained 1–4 isolates, from up to two different participants (Fig. 4). Three of these six LTCF clusters contained closely associated CUH isolates. For each of these three clusters we calculated the pairwise SNP differences between the LTCF and CUH isolates in the same cluster, which were in the ranges of 7–66, 19–67 and 10–11 SNPs, respectively.

The 251 ST131 isolates were further categorised by defining the *fimH* allele, which demonstrated that 194 carried the *fimH30* allele, placing them in clade C [57]. The remaining 57 isolates carried the following *fimH* alleles: H1 (n = 3), H12 (n = 2), H17 (n = 30), H25 (n = 2), H6 (n = 19) and unknown (n = 1). Clade C has been further divided into three sub-clades termed C0, C1 and C2 based on point mutations [58–60]*.* The sub-clades of clade C were determined in this collection and are shown in Fig. 4, together with information on the mutations identified in Additional file 1.

## Discussion

Prolonged or repeated antimicrobial use is a driver for the emergence of antimicrobial resistance and is an established risk factor for ESBL carriage [61–63]. We found that study participants who carried ESBL *E. coli* had been prescribed significantly more antimicrobials than those who did not. Residence in a LTCF is also a known risk factor for faecal carriage of ESBL *E. coli* [61]. In our six-month study, 38% of participants carried ESBL *E. coli*, which is consistent with carriage rates identified in previous studies from the UK [28, 29] and internationally [31, 64–66].

Serial sampling allowed us to describe within-host diversity of the same and different clades and lineages. WGS of healthcare-associated pathogens has begun to delineate the potential for complex within-host diversity [67–69]. To date, a small number of studies have investigated within-host diversity of ESBL *E. coli* using WGS [70, 71]. Sequencing of 16 bacterial colonies isolated from single stool samples obtained from eight children presenting to a hospital in Cambodia identified within-host carriage of multiple *E. coli* lineages and variation in virulence and antibiotic resistance genes [70], supporting the findings in our study. Bayesian estimation of substitution rates in ST131 gave a value of ~1 SNP per genome per year [72]. The median level of within-host diversity in LTCF residents was four SNPs, which is consistent with long-term carriage and within-host evolution. We also analysed the relationship between ESBL *E. coli* and non-ESBL *E. coli* within individuals. Some participants carried ST131 ESBL *E. coli* and non-ESBL *E. coli* that were indistinguishable at the core genome level. This is consistent with previous studies that described loss and acquisition of either *bla*_CTX-M-15_ or the ESBL plasmid within an ST131 population and the presence of insertion and transposon sequences flanking the upstream and downstream regions of *bla*_CTX-M-15_ [72, 73].

The dominant ESBL *E. coli* lineage identified in our study was ST131, a finding consistent with previous studies [74–76]. We also isolated ST38 ESBL *E. coli* from one participant, which was acquired from an unknown reservoir during the study period. ST38 is commonly identified among human carriage and invasive isolates [76–78] and is increasingly being associated with *bla*_OXA-48_, a beta-lactamase gene with significant carbapenamase activity, although this was not identified here [79, 80].

An important observation in this study was the value of contextual genetic databases in defining the relationship of ST131 isolates between study participants. Analysis of ST131 LTCF isolates alone suggested that participants carried several related but distinct populations, with three groups of participants carrying distinct clones of the same lineage. However, placing ST131 LTCF isolates into the genetic context of local and national ST131 collections revealed that the majority of isolates from LTCF participants clustered together, indicating acquisition of ST131 ESBL *E. coli* from a local lineage or a shared reservoir predating the study. The remaining LTCF isolates formed genetically distinct clades, indicating multiple introductions to the LTCF.

Our study had a number of limitations. A comprehensive understanding of carriage and transmission patterns requires 100% data capture, but we were only able to recruit 50% of the LTCF residents. Furthermore, our study design did not include sampling of healthcare workers, family members or the environment, all of which are potential donors or recipients of ESBL *E. coli*.

## Conclusions

We confirmed that residents of a LTCF were a reservoir for multidrug-resistant *E. coli* and that ST131 dominated in this setting. We found evidence for a shared reservoir for ST131 within the LTCF, and between the LTCF and a nearby acute hospital. This suggests putative transmission within this broader healthcare network and underlines the importance of the interconnectivity in the spread of multidrug-resistant pathogens.

## Additional file

Additional file 1: Additional information for the dataset used in this study. The additional file includes demographic information including location of isolation and sample type along with genome sequence QC data, results of genomic typing and screening for antimicrobial resistance genes. (XLSX 145 kb)

## Abbreviations

BSAC: British Society for Antimicrobial Chemotherapy
CUH: Cambridge University Hospitals NHS Foundation Trust
ECDC: European Centre for Disease Prevention and Control
ESBL: Extended-spectrum beta-lactamase
IQR: Interquartile range
LTCF: Long-term care facility
MALDI-TOF: Matrix-assisted laser desorption/ionization time-of-flight mass spectrometry
MGE: Mobile genetic element
MLST: Multi-locus sequence type
PCR: Polymerase chain reaction
SNP: Single nucleotide polymorphism
ST: Sequence type
UK: United Kingdom
UPEC: Uropathogenic *E. coli*
WHO: World Health Organization
